# A Label-Free Electrochemical Genosensor for the Rapid Detection of *Campylobacter jejuni*, *C. coli*, *C. lari* and *C. upsaliensis*

**DOI:** 10.3390/mi17040457

**Published:** 2026-04-08

**Authors:** Priya Vizzini, Rosanna Toniolo, Rossella Svigelj, Fabiola Zanette, Marisa Manzano

**Affiliations:** Dipartimento di Scienze AgroAlimentari, Ambientali e Animali, Università degli Studi di Udine, 33100 Udine, Italyrossella.svigelj@uniud.it (R.S.); zanette.fabiola@spes.uniud.it (F.Z.)

**Keywords:** screen-printed gold electrodes (SPAuEs), differential pulse voltammetry, *Campylobacter* spp., food safety, DNA probe

## Abstract

*Campylobacter* spp. is one of the most common pathogens responsible for gastroenteritis in developed countries and is raising public health concerns worldwide. This work optimized a label-free electrochemical genosensor based on screen-printed gold electrodes (SPAuEs) for the rapid detection of *Campylobacter jejuni*, *C. coli*, *C. lari* and *C. upsaliensis*. SPAuEs were functionalized with a specific thiolated DNA probe and tested with a ferrocyanide solution for signal production. The optimization of the conditions was obtained using DNA extracted from pure cultures of *Campylobacter* spp. and negative controls such as *Escherichia coli*, *Listeria innocua*, *Salmonella* spp., and *Helicobacter pylori*. Cyclic voltammetry (CV) and differential pulse voltammetry (DPV) were compared to assess sensitivity and specificity. The relative change in intensity of the ferrocyanide anodic peak (Ipa) was proportional to the value of *Campylobacter* spp. DNA concentrations in the range of 1 pg/µL to 10^4^ pg/µL. The limit of detection of our optimized system was 1.06 pg/μL. After optimization, the method was applied to chicken meat samples from the market. The proposed electrochemical DNA biosensor was able to detect *Campylobacter jejuni*, *C. coli*, *C. lari* and *C. upsaliensis* after selective enrichment and DNA isolation within 60 min of DNA extraction, demonstrating its usefulness for routine analyses.

## 1. Introduction

Bacteria, viruses, or parasites can spread to humans through direct contact, food, water, and the environment. In Europe, over 23 million cases of bacterial zoonoses are reported annually by the World Health Organization [1], of which according to the EFSA (European Food Safety Authority) and ECDC (European Centre for Disease Prevention and Control) reports [2], the most common cause of food-borne zoonotic disease is *Campylobacter* spp. Campylobacteriosis is primarily caused by *C. jejuni*, *C. coli*, *C. lari*, and *C. upsaliensis* after consumption of undercooked contaminated chicken meat. The amount of chicken meat available in retail markets containing *Campylobacter* spp. in the EU ranges between 60 and 80%, whereas in the US it is up to 98%. The EFSA estimated around 2.4 billion € per year as the cost of campylobacteriosis to public health as a consequence of hospitalization costs, work absence, financial losses associated with consumer concerns about food quality, and legal proceedings due to food contamination [3].

To reduce costs and improve food safety, effective measures for food handling along the entire farm-to-fork chain must be complemented by rapid and reliable analytical methods. The official plate count method (ISO 10272:2017) [4] requires approximately one week for the detection of *Campylobacter* spp., which limits timely decision-making. In contrast, PCR-based molecular techniques significantly reduce analysis time to 24–48 h, enabling faster intervention and risk management; however, these methods may suffer from drawbacks such as inhibition of DNA polymerase by contaminants present in food samples [5,6]. Immunoassay methods rely on *Campylobacter* growth on selective agar plates; thus, they are labor-intensive and time-consuming. Moreover, samples must be delivered to the laboratory for analyses carried out by highly qualified personnel. To help food industries in processing huge numbers of samples in a short time while maintaining specificity and sensitivity, various biosensors with specific DNA probes have been utilized in recent years for the detection of pathogenic microorganisms [7,8,9,10,11]. A recent trend involves the development of electrochemical biosensors that meet the food industry’s demands, as they are rapid, specific and simple to use and miniaturize [12]. Genosensors, which use a single-stranded (ss)DNA probe as a bioreceptor for the detection of DNA targets in samples, can produce a signal in a label-free manner through binding between the DNA target and the bioreceptor (capture DNA probe). The signal is measured and recorded by the detector. Moreover, the DNA probes bind target DNA with the advantage of the non-hazardous nature of DNA compared to viable pathogenic cells, which can represent a great risk for the operator and require the implementation of procedures for hazardous waste handling and disposal. In recent years, screen-printing (thick film) technology, applied to biosensor construction, has been considerably improved [13,14,15,16,17,18,19]. Indeed, the use of a screen or mesh for the layer-by-layer deposition of ink upon a solid substrate defines the geometry and area of the sensor through an automatic process. This provides good reproducibility of the biosensor. The small size of the biosensors enables connection to portable instrumentation allowing on-site detection of target analytes, as the biosensors are cheap, disposable and simple to use.

Many factors, such as the method of fabrication, the properties of the materials used, and the properties of the surface, influence the performance of the sensors. Hence, the sensitivity and selectivity of screen-printed electrodes (SPEs) is constantly improving. Gold electrodes have been found extremely useful for their favorable electron transfer kinetics, relatively good stability, chemically inert nature, and excellent resistance to oxidation, and for the possibility of using ssDNA probes for Au binding [20].

The aim of this work was to apply a label-free specific and sensitive electrochemical biosensor to detect *Campylobacter jejuni*, *C. coli*, *C. lari* and *C. upsaliensis* in chicken samples to reduce the time required for pathogen detection by immobilizing a specific thiolated DNA probe on screen-printed gold electrodes (SPAuEs). The specificity of the probe was verified using the dot-blot enzyme immunoassay; moreover, the screen-printed tests were compared to the official ISO method.

## 2. Materials and Methods

### 2.1. Equipment and Reagents

The screen-printed gold electrodes (SPAuEs) DropSens 220 AT (Metrohm-DropSens, Origgio, Italy) were used. The electrochemical cell consisted of a gold working electrode (WE) (4 mm in diameter), a gold counter electrode (CE), and a silver reference electrode (RE). Sulfuric acid (H_2_SO_4_) (Carlo Erba, Milan, Italy) at 0.5 M (96% sulfuric acid) and phosphate buffer saline (PBS) (Sigma-Aldrich, Milan, Italy) were used. 6-Mercapto-1-hexanol (MCH) at 1 mM (Sigma-Aldrich, Milan, Italy) was prepared and used as a blocking solution on the gold (Au) electrode, while a solution of potassium ferrocyanide (K_4_[Fe(CN)_6_] 10 mM) prepared using sterilized PBS buffer 1X (AnalytiCals, Milan, Italy) was used as a redox probe.

The electrochemical analysis was conducted using a PGSTAT101 Autolab potentiostat galvanostat and NOVA 2.1 software (Metrohm-Autolab B.V., Utrecht, The Netherlands).

### 2.2. Bacterial Strains

The bacterial strains used for the work are listed in Table 1. *Campylobacter* strains were cultured in Brain–Heart Infusion (BHI) broth (Oxoid, Milan, Italy), under micro-aerophilic conditions generated with a Sachet Oxoid™ CampyGen™ 2.5 L (Oxoid, Italy) at 37 °C for 48 h, then isolated on Columbia blood agar by streaking twice and incubated under the same conditions.

All strains used as negative controls were cultured at 37 °C on BHI agar, in aerobic conditions, except for *C. fetus*, *Arcobacter butzleri*, and *Helicobacter pylori* which were grown in a micro-aerophilic atmosphere [21]. The selective media Palcam Agar Base and XLD medium (Oxoid, Italy) were used for *Listeria monocytogenes* and *Salmonella* spp., respectively. All strains were checked by Gram stain, cell morphology, and oxidase and catalase tests.

### 2.3. Probe and Primer Design

The DNA probe CampyP3 5′- TAG TGG CGC ACG GGT GAG TAA GGT ATA GTT AAT CTG -3′ (patents: it 102020000012496 and fr 20 05578 2020) was designed on the 16S rRNA gene that encodes ribosomal RNA to detect the major species responsible for gastroenteritis: *C. jejuni*, *C. coli*, *C. lari*, and *C. upsaliensis*.

The sequences were downloaded from GenBank and used for alignment with “Multiple sequence alignment with hierarchical clustering” [22] (Table A1).

The primers CampyPFw and CampyPRv were designed within the intergenic spacer (ITS) 16S-23S gene using the sequences GQ167702.1 for *C. jejuni*, GQ167720.1 for *C. coli*, AB644222.1 for *C. lari*, and DQ871249.1 for *C. upsaliensis* as described by Vizzini et al. [10].

The features of the probe were tested in silico using the software OligoAnalyzer 3.1 (https://eu.idtdna.com/calc/analyzer, accessed 14 March 2019) and the specificity was verified by Fast PCR 6.1, and Blast [23] using both positive and negative control sequences (Table A1). Primers and probe were synthetized by Eurofins Genomics (Milan, Italy).

Additionally, a sequence complementary to the CampyP3 probe (CCP3) was used as a positive control for the assay.

### 2.4. Probe Labeling

For application in the dot-blot enzyme immunoassay, the CampyP3 probe was labeled at the 5′ end with digoxigenin (CampyP3-Dig) and for the voltametric application by adding a thiol group at the 5′ end (CampyP3-SH assay).

The CampyP3-SH probe was activated according to the manufacturer protocol (Eurofins Genomics, Ebersberg, Germany) and standardized at 10 ng/μL in PBS buffer before immobilization on the gold surface of the SPAuEs.

### 2.5. Food Samples ISO 10272:2017 Analysis

**Figure 1:**
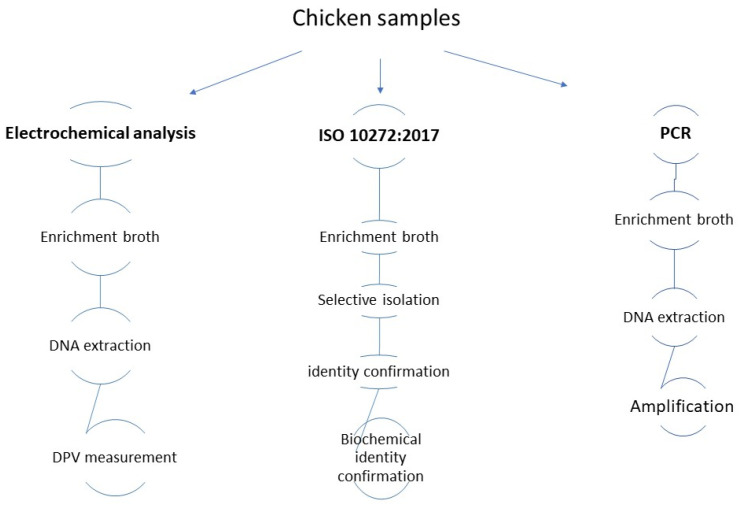
Workflow of the experiments performed.

Three chicken samples (1C, 2C, and 3C) were purchased at butcher shops in Italy. *Campylobacter* detection was performed according to the official ISO 10272:2017 method. From each sample, 10 g of chicken meat with skin was added to 90 mL of Bolton broth (Oxoid, Milan, Italy) and incubated in micro-aerophilic conditions for 40–48 h at 41.5 °C [24]. After enrichment, for selective isolation, a loop (10 µL) of the broth was streaked on the two selective media mCCDA and Skirrow (Oxoid, Milan, Italy), and plates were incubated for 48 h at 41.5 °C in micro-aerophilic conditions before being examined for the presence of suspected colonies. Confirmation was carried out from 1 to 5 suspected colonies per plate by streaking on two blood agar base plates, of which, one was incubated at 41.5 °C and one at 25 °C for 48 h. *Campylobacter* spp. is not able to grow at 25 °C; therefore the absence of growth at 25 °C was expected, except for *C. fetus*. The identity confirmation includes an oxidase test and motility test, carried out using Brucella broth (Thermofisher Scientific, Monza, Italy). DNA was extracted from the enrichment broth after 40–48 h. Figure 1 reports the workflow of the analyses.

### 2.6. DNA Extraction from Pure Culture and Food Samples

Genomic DNA from bacterial pure cultures and food samples was extracted using a slightly modified version of a previously described protocol [10].

Briefly, bacterial colonies were collected from agar plates and suspended in tubes containing glass beads (0.5 mm diameter) and 300 μL of breaking buffer (Triton2%, SDS1%, NaCl 100 mM, Trizma-Base at pH 8 (Sigma, Milan, Italy).

For food samples, 2 mL from the enrichment broth was centrifuged for 10 min at 10,000× *g* and resuspended in tubes containing breaking buffer and glass beads [25].

The DNA was treated with RNAse at 37 °C for 1 h and subjected to measurement using a spectrophotometer Nanodrop™ 2000C (ThermoFisherScientific, Milan, Italy) to verify purity and concentration and stored at −20 °C before utilization.

### 2.7. CampyP3 Probe Dot-Blot Enzyme Immunoassay

Aliquots of 1 µL of DNA samples were treated at 95 °C for 10 min, spotted on a positively charged nylon membrane (Biorad, Milan, Italy) and immobilized under UV light at 254 nm for 10 min. The hybridization step was conducted overnight at 63 °C in the Dig Easy Hyb buffer (Roche, Milan, Italy) adding the CampyP3-Dig probe at 0.1 ng/mL during the final shakings. All subsequent steps were conducted as reported in [26].

### 2.8. Functionalization of SPAuEs and Electrochemical Measurements

SPAuEs were electrochemically cleaned using cyclic voltammetry in a 0.5 mol/L sulfuric acid solution (Carlo Erba, Milan, Italy) varying the potential from 0.0 to +1.3 V ten times at a scan rate of 100 mV/s.

Subsequently, the working electrode was functionalized using 12 µL of the CampyP3-SH probe at a final concentration of 10 ng/µL in PBS (Sigma-Aldrich, Milano, Italy), after denaturation at 95 °C for 10 min, obtaining a concentration of 9.55 ng/mm^2^ which was incubated at 25 °C overnight. After washing the surface twice with 1X PBS, the gold electrode surfaces were blocked with 12 µL of mercaptohexanol (MCH) 1 mM in PBS and incubated for 1 h at 25 °C, obtaining a concentration of 12.420 ng/mm^2^. After surface functionalization, the screen-printed gold electrode was rinsed with 1× PBS, dried at room temperature, and subsequently used for target DNA hybridization and electrochemical measurements.

The hybridization of DNA extracted from microorganisms used as positive and negative controls and DNA extracted from food samples was conducted for 1 h at 63 °C. The sequence complementary to the Campy P3 probe was used as a positive control for the tests.

DNA samples were diluted in PBS and then denatured at 95 °C for 10 min before being used for hybridization. After each functionalization and measurement step, the SPAuEs were rinsed twice with 500 µL sterile water and dried under a hood.

Cyclic voltammetry (CV) and differential pulse voltammetry (DPV) measurements were conducted using 10 mM potassium hexacyanoferrate (II) K_4_[Fe(CN)_6_] in PBS after functionalization and hybridization steps.

CV measurements were performed from −0.2 V to +0.6 V at a scanning rate of 0.1 V/s. DPV measurements were performed from −0.2 V to +0.4 V using the following parameters: scan rate 0.01 V/s, interval time 0.5 s, modulation time 0.05 s, modulation amplitude 0.15 V, step 0.005 V.

For each sample, the anodic peak current recorded by CV and DPV was measured and the percentage signal suppression after hybridization (%SS) was calculated using the following equation:

%SS = [(Ipa blank − Ipa samples)/Ipa blank] × 100

where Ipa blank represents the K_4_[Fe(CN)_6_] anodic current recorded before hybridization and Ipa sample represents the current after hybridization.

After verifying the specificity of the CampyP3 probe towards the microorganisms listed in Table 1, the DNA of *E. coli* was used as the negative control for the tests conducted with the genosensor.

The same protocol was used to analyze chicken meat samples.

### 2.9. PCR Protocol

PCRs were carried out on chicken samples naturally contaminated using the CampyPFw and CampyPRv primers in a reaction mixture containing the following reagents: 5 µL of AmpliTaq Buffer (1.5 mM) 1 µL of PCR Nucleotide Mix dNTPs (10 mM each dNTPs), 1 µL of each primer (10 μM), 0.25 μL AmpliTaq DNA polymerase (5 units/µL) and 1 µL of DNA extracted standardized at 100 ng/µL in a final volume of 50 μL.

Thermal cycler (C1000 Touch^TM^, Bio-Rad, Milan, Italy) conditions consisted of: 95 °C denaturation for 5 min; 30 cycles of 95 °C for 1 min, 58 °C for 30 s, 72 °C for 30 s and a final extension at 72 °C for 7 min. Then, 5 μL PCR product was mixed with 5 μL Gel loading Buffer 1X and electrophoresed in 1.5% agarose gel Tris-borate-EDTA buffer 0.5X (Sigma-Aldrich, Milan, Italy) stained with a final concentration of 0.5 μg/mL ethidium bromide (Sigma- Aldrich, Milan, Italy).

A 100 bp ladder (Promega, Milan, Italy) was used to size products. The electrophoretic run was performed at 120 V for 40 min. The results were examined under UV light in a cabinet (BioImaging System GeneGenius, Syngene, Cambridge, UK). All reagents were purchased from Applied Biosystem (Monza, Italy).

## 3. Results

### 3.1. Specificity and Sensitivity of the CampyP3 Probe with Dot-Blot Enzyme Immunoassay

The CampyP3 probe tested with the dot-blot enzyme immunoassay on DNA from the bacteria listed in Table 1 showed specificity for *C. jejuni*, *C. coli*, *C. lari*, and *C. upsaliensis* as predicted in silico with the test performed using multiple sequence alignment with hierarchical clustering [22], Amplifix 1.7.0, OligoAnalyzer3.1 and FastPCR6.1 software. Table 2 reports the results of the tests conducted using as a template DNA from the positive and negative controls. The presence of blue spots indicates the hybridization of the CampyP3 probe to the DNA used as a target (positive result, +), while the absence of the blue spots indicates that the probe did not hybridize to the target DNA used and it was considered a negative result (-).

The sensitivity of the test was conducted using as a template the sequence complementary to the CampyP3 probe (CCP3) from 100 ng/μL to 0.0001 ng/μL and gave positivity until 0.1 ng/μL of the target (Table 2).

### 3.2. Electrochemical Biosensor Performance

Sensitivity test

The genomic DNA of *C. jejuni* was diluted at 1000 pg/μL, 100 pg/μL, 10 pg/μL, and 1 pg/μL and hybridized to the CampyP3 probe immobilized on SPAuEs at 10 ng/μL. The means of three measurements $\left(\bar{I_{Pa}}\right)$ of the voltammetric response, CV and DPV, are reported in Table A2. The differences between the anodic peak current recorded before hybridization (Ipa blank) and the anodic peak current recorded after hybridization (Ipa samples) for CV (Δ Ipa CV) and DPV (Δ Ipa DPV), as well as the standard deviation (SD) of Δ Ipa and %SS, are reported in Table A2.

**Figure 2:**
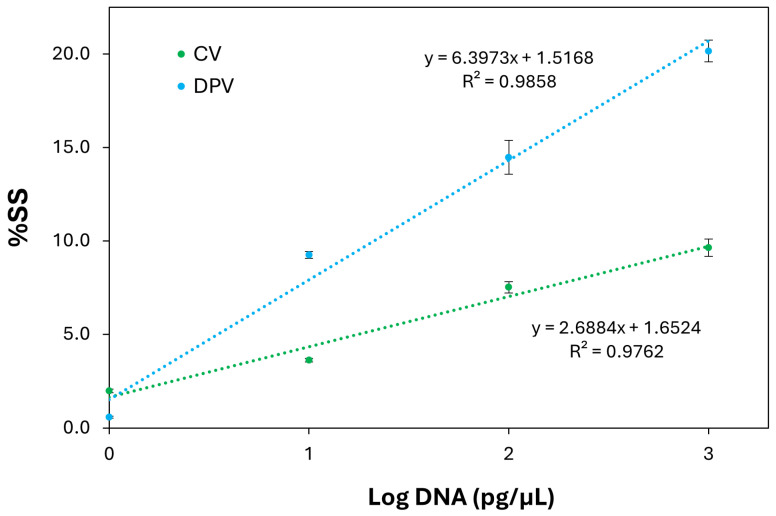
Calibration curve obtained by plotting the percentage signal suppression (%SS) on the *Y*-axis versus the logarithm of the *C. jejuni* genomic DNA concentration (expressed in pg/μL) on the *X*-axis. %SS values were obtained using measurements from CV and DPV. The error bars represent the standard deviation calculated from three independent sets of measurements performed using modified SPAuEs on different days.

**Figure A1:**
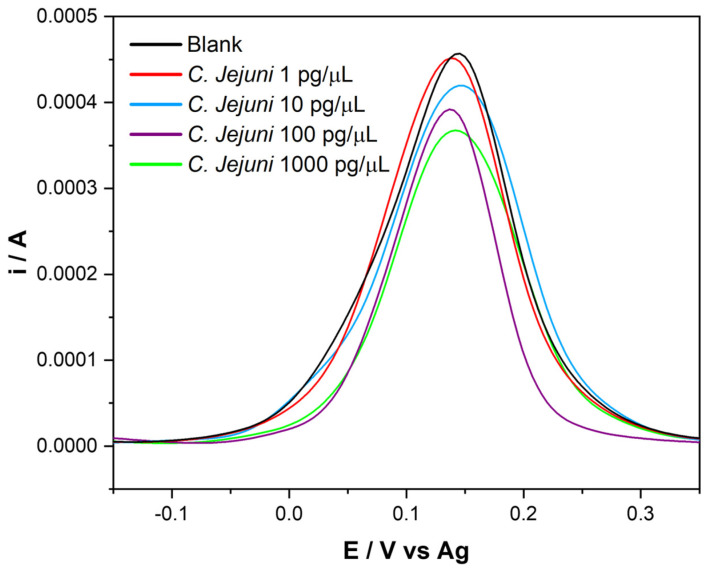
Differential pulse voltammetry curve for samples of DNA of *C. jejuni* DSM 4688 in the range from 1 pg/μL to 1000 pg/μL.

The calibration curves of *C. jejuni* by CV and DPV shown in Figure 2 were obtained by plotting %SS against the logarithmic concentration of the target DNAs expressed in pg/μL, obtaining the following linear equations: y = 2.6884 ± (0.2971)x + 1.6524 ± (0.5557) (R^2^ = 0.9762) for CV and y = 6.3973 ± (0.5437)x + 1.5168 ± (1.0171) R^2^ = 0.9858) for DPV. In Figure A1, the DPV curves for the DNA of *C. jejuni* DSM 4688 are reported ranging from 1 pg/μL to 1000 pg/μL.

Based on the DPV calibration equation above reported and the standard deviation of %SS at the lowest *C. jejuni* DNA concentration, a limit of detection (LOD) of 1.06 pg/µL (for a signal-to-noise ratio of 3) and a limit of quantification (LOQ) of 3.52 pg/µL (for a signal-to-noise ratio of 10) were determined.

Despite the limited number of concentration points (1, 10, 100, and 1000 pg/µL), the DPV calibration curve showed good linearity over the tested range, with a coefficient of determination close to 0.99 (R^2^ = 0.9858), indicating a consistent relationship between the DNA concentration and the electrochemical signal. The low dispersion of the experimental points around the regression line further supports the reliability of the observed trend.

Selectivity test

**Figure 3:**
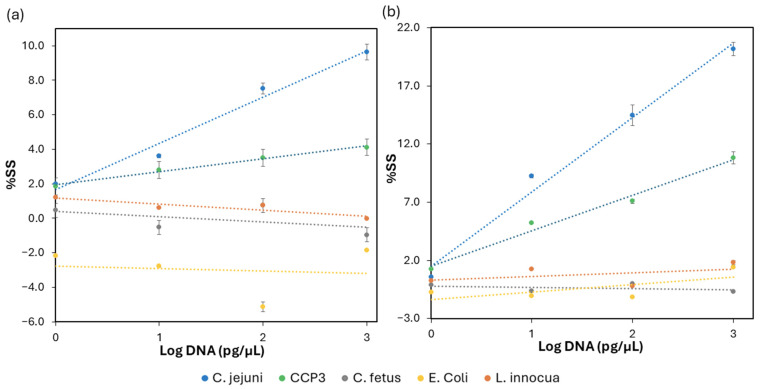
Selectivity test. Calibration curve obtained by plotting the percentage of suppression signal (%SS) on Y-axis against log DNA of *C. jejuni* DSM 4688, CCP3, *C. fetus* DSM 5361, *E. coli* DI4A, *L. innocua* DSM 20649 on X-axis expressed as Log pg/µL. %SS values were obtained using measurements from cyclic voltammetry (CV) (**a**) and differential pulse voltammetry DPV (**b**). The error bars represent the standard deviation calculated from three independent sets of measurements performed using modified SPAuEs on different days.

The selectivity of the assay was tested using as a target the short sequence CCP3 which matches perfectly with the CampyP3 probe and the genomic DNA of *C. jejuni* as positive controls. Negative controls included the DNA of *C. fetus,* for the high similarity of the whole genome with *C. jejuni*; *L. innocua*, as a Gram-positive bacterium; and *E. coli* (Gram-negative) because it is usually present in chicken samples. For CCP3 and each bacterium, several DNA concentrations from 1 to 10^4^ pg/μL were used for hybridization to the working electrode of the SPAuEs functionalized with the probe CampyP3 at 10 ng/μL. The measurements were processed as described in the sensitivity test paragraph and the data are reported in Table A2. Calibration curves for positive and negative controls were obtained by plotting %SS against the logarithmic concentration of the target DNAs expressed in pg/μL for both CV and DPV modes (Figure 3). The data show a slight increase in %SS for CCP3, as expected for the minor steric hindrance, in comparison to the values obtained for *C. jejuni* genomic DNA.

Negative controls were characterized by the following linear equations: *C. fetus* y = −0.3046x + 0.3844 (R^2^ = 0.2378) by CV and y = −0.1132x − 0.1922 (R^2^ = 0.1676) by DPV; *E. coli* y = −0.1328x − 2.7905 (R^2^ = 0.0133) by CV and y = 0.6305x − 1.3459 (R^2^ = 0.4579) by DPV; and *L. innocua* y = −0.3526x + 1.1658 (R^2^ = 0.8046) by CV and y = 0.3184x + 0.3024 (R^2^ = 0.1997) by DPV.

All of the negative controls were tested at the same concentrations used for the positive controls (Table A2); the maximum value obtained for the negative controls was 1.8%SS for *L. innocua* at 1000 pg/µL by DPV.

These results show no proportional increment in the %SS and the low R^2^ of the linear equations for negative controls, compared to the increase in %SS and the good R^2^ of *Campylobacter,* which confirms the specific hybridization of the probe only to the DNA of the target *C. jejuni*.

In addition, small negative %SS values for non-target DNA are observed at concentrations higher than the sensor LOQ. These minor deviations are attributable to small electrochemical baseline fluctuations or experimental variability. Moreover, for concentrations above the LOQ, the %SS values for target DNA remain consistently positive, reproducible, and clearly distinguishable from non-target responses.

Based on the comparison between cyclic voltammetry (CV) and differential pulse voltammetry (DPV), the latter exhibited higher sensitivity attributable to its ability to minimize background current and reduce capacitive contributions. Therefore, DPV was selected for the analysis of chicken samples.

### 3.3. Official and Electrochemical Food Analysis

The analyses carried out on the chicken samples (1C, 2C, and 3C) with both the official ISO 10272:2017 and the PCR methods confirmed the presence of *Campylobacter* spp. in samples 1C and 2C.

The %SS values of samples 1C, 2C, and 3C analyzed in DPV mode using 12 μL of DNA diluted at 1:10 hybridized on the gold functionalized surface of the working electrode of the SPAuEs were 16.81%, 12.16%, and 1.59%, respectively, while the value of %SS of *E. coli* at 1 ng/µL was −4.04%. The concentration of DNA of *C. jejuni* (expressed in pg/µL) in samples 1C, 2C, and 3C was calculated using the linear equation y = 6.3973 ± (0.5437)x + 1.5168 ± (1.0171) R^2^ = 0.9858) of the DPV regression line of *C. jejuni.*

**Figure 4:**
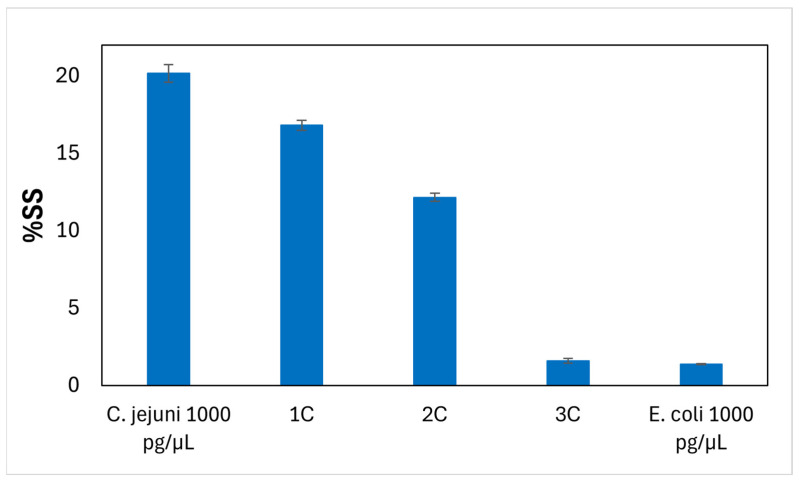
Percentage of suppression signal (%SS) of chicken samples (1C, 2C and 3C) and *C. jejuni* DSM 4688 (positive control) and *E. coli* DSM 24155 obtained by differential pulse voltammetry (DPV). The error bars represent the standard deviation calculated from three independent sets of measurements performed using modified SPAuEs on different days.

Samples 1C and 2C showed SS% values useful for calculating the corresponding amounts of DNA of *Campylobacter,* which resulted in 205.22 pg/µL and 38.37 pg/µL, respectively; thus, these samples were considered positive for the presence of *Campylobacter* spp. The %SS of sample 3C brings about a value of 0. 85 pg/µL of DNA, below the range of detection of the assay, suggesting that the sample was likely negative for the presence of *Campylobacter* (Figure 4).

## 4. Discussion

The tests performed on DNA from the positive and negative controls highlight the specificity of the probe, which produced blue spots due to hybridization only for the bacteria considered as positive and listed in Table 1. These good results allowed the utilization of CampyP3 during application of the genosensor using the DPV mode for the testing of chicken samples.

The suppression signals (%SS) produced by the hybridization between the CampyP3 probe and the target showed the sensitivity and selectivity of the electrochemical biosensor for *C. jejuni*. Indeed, the increment of %SS is proportional to the increment of the DNA concentration hybridized.

The biosensor produces a signal from hybridization with CCP3, a short complementary sequence to CampyP3 probe, but the %SS showed less sensitivity due to its smaller steric hindrance compared to the genomic DNA of *C. jejuni*, as expected.

From the data reported, the DPV method is more sensitive than CV; indeed, the slope of the calibration curves from both CCP3 and *C. jejuni* is greater for DPV than CV. This means a greater sensitivity of the assay to discriminate positive controls from negative controls. Moreover, the calibration curve obtained for DPV showed a better R^2^ value of 0.9858 compared with the R^2^ value of 0.9762 for the CV mode.

Recent advances in biosensing technologies highlight the development of highly sensitive and portable detection platforms. For example, Chen et al. [27] reported a DNA nanomachine-based fluorescence analyzer, Jiang et al. [28] reviewed intelligent sensing approaches for food safety, and Cai et al. [29] developed a multiplexed graphene-based immunosensor, all demonstrating improved sensitivity and analytical performance.

Compared to these systems, our approach focuses on a simple and cost-effective electrochemical genosensor for *C. jejuni* detection in food matrices. While some platforms achieve ultra-low detection limits through complex designs, our method provides a practical balance between sensitivity, specificity, and operational simplicity, making it suitable for routine analysis.

Several studies on DNA detection systems for *Campylobacter* spp. using as bioreceptor a DNA probe [7,10,30,31,32] report limits of detection (LODs) of 90 pM, 20 pM, 0.33 ng/µL, 1 ng/µL and 3 pg/µL, in our case the LOD was 1.06 pg/µL, demonstrating competitive sensitivity within the context of existing electrochemical genosensors. This improved sensitivity, combined with the simplicity of the method, indicates the potential applicability of our protocol in routine analyses and its ability to reduce the time required for *Campylobacter* detection in chicken meat.

## 5. Conclusions

The electrochemical biosensor proposed in this work can be a good alternative to the classical plate-based methods for the detection of *Campylobacter* spp. in poultry meat. It is sensitive—in fact, it reaches a LOD of 1.06 pg/μL, lower than other methods; it is simple to use; and it is rapid—in fact, it is possible to obtain a result within 60 min from DNA extraction while the ISO method requires days. The developed genosensor represents a promising tool for food analyses. The next step to make the method useful for in situ application will be the optimization of DNA extraction without the utilization of bench devices and the need for trained personnel.

## Figures and Tables

**Table 1:** List of bacteria used in specificity tests.

Controls	N.	Microorganisms	Collection Code
Positive	1	*Campylobacter jejuni* subsp. *jejuni*	DSM 4688 ^A^
	2	*C. coli*	DSM 24155
	3	*C. lari* subsp. *lari*	DSM 11375
	4	*C. upsaliensis*	DSM 5365
Negative	5	*C. fetus*	DSM 5361
	6	*Helicobacter pylori*	DSM 7492
	7	*Arcobacter butzleri*	DSM 8739
	8	*Listeria innocua*	DSM 20649
	9	*Salmonella enterica*	DSM 9378
	10	*Escherichia coli*	DI4A ^B^

^A^ DSM: Deutsche Sammlung von Mikroorganism und Zellkulturen GmbH (Braunschweig, Germany) and ^B^ DI4A: Department of Agricultural, Food, Environmental and Animal Sciences (University of Udine, Udine, Italy).

**Table 2:** Sensitivity of the CampyP3 DNA probe tested with dot-blot on the sequence complementary to the probe (CCP3) and specificity of the probe obtained testing on DNA from various microorganisms.

Sample	DNA Expressed in ng/μL	Presence/Absence of the Bleu Spots	Results *
Sensitivity of the CampyP3 DNA Probe			
CCP3	100	Presence	+
CCP3	50	Presence	+
CCP3	10	Presence	+
CCP3	1	Presence	+
CCP3	0.1	Presence	+
CCP3	0.01	Absence	-
CCP3	0.001	Absence	-
CCP3	0.0001	Absence	-
Sensitivity of the CampyP3 DNA Probe			
*Escherichia coli* DI4A	100	Absence	-
*Helicobacter pylori* DSM 7492	100	Absence	-
*Arcobacter butzleri* DSM 8739	100	Absence	-
*Salmonella enterica* DSM 9378	100	Absence	-
*Campylobacter fetus* DSM 5361	100	Absence	-
*Campylobacter jejuni* DSM 4688	100	Presence	+
*Campylobacter coli* DSM 24155	100	Presence	+
*Campylobacter lari* DSM 11375	100	Presence	+
*Campylobacter upsaliensis* DSM 5365	100	Presence	+

* The presence of the blue spot means that the probe hybridized the target DNA, and the result is considered positive; the absence of the blue spot means that the probe did not hybridize the target DNA, and the result is considered negative.

## Data Availability

The data presented in this study are available on request from the corresponding author. The data are not publicly available due to privacy.

## Appendix A

**Table A1:** Accession numbers of 16S and 16S-23S sequences of *Campylobacter* species and other bacteria used for the design of the probe.

Accession Number Bacteria		
1	NR_117760.1	*Campylobacter jejuni*
2	GQ167702.1	*C. jejuni*
3	JX912505.1	*C. coli*
4	GQ167720.1	*C. coli*
5	NR_117762.1	*C. lari*
6	AB644222.1	*C. lari*
7	KP064555.1	C. *fetus*
8	GQ167709.1	*C. concisus*
9	NR_115289.11	*C. upsaliensis*
10	DQ871249.1	*C. upsaliensis*
11	JX912515.1	*C. gracilis*
12	L04322.1	*C. concisus*
13	AB301966.1	*C. fetus* subsp. *fetus*
14	M65011.1	*C. fetus* subsp. *venerealis*
15	NZ_FPBB01000005.1	*C. hyointestinalis*
16	NZ_jhqq01000009.1	*C. mucosalis*
17	nzNZjmti01000045.1	*C. sputorum biovar sputorum*
18	EF373994.1	*Aeromonas sobria*
19	AM062666.1	*Bacillus cereus*
20	EF205020.1	*B. subtilis*
21	AF047423.1	*Citrobacter freundii*
22	EF527445.1	*Escherichia coli*
23	FJ410387.1	*Enterobacter aerogenes*
24	AF047426.1	*E. aerogenes region*
25	EU078570.1	*E. cloacae*
26	AY277975.1	*Helicobacter ganmani*
27	DQ399570.1	*Klebsiella pneumoniae*
28	AB092638.1	*Lactiplantibacillus plantarum*
29	AF000655.1	*Legionella pneumophila*
30	AB295116.1	*Leuconostoc lactis*
31	AY684791.1	*Listeria monocytogenes*
32	AB089244.1	*Morganella morganii*
33	AF405374.1	*Pediococcus pentosaceus*
34	FJ518598.1	*Proteus vulgaris*
35	JN418884.1	*Pseudomonas aeruginosa*
36	AF268968.1	*Ps. brennerii*
37	AM086254.1	*Ps. brennerii*
38	EF198908.1	*Ps. Fluorescens*
39	KF857261.1	*P. migulae*
40	AF046822.1	*Salmonella enterica*
41	KX696458.1	*Shigella sonnei*
42	U11784.1	*Staphylococcus aureus*
43	AY531067.1	*Vibrio parahaemolyticus*
44	FJ429988.1	*Weissella cibaria*
45	AF293850.1	*Yersinia enterocolitica*

**Table A2:** Measurement of the average anodic peak current ($\bar{\mathrm{Ipa}}$) standard deviation (SD), the ΔIpa and the percentage of suppression signal (%SS) by CV and DPV of CCP3, *C jejuni*, *C. fetus*, *E. coli*, and *L. innocua*. The Ipa values are expressed in microampere (μA). Three repetitions for each mean result were conducted.

		CV				DPV			
Sample DNA Concentration (pg/µL)	Log DNA	$\bar{I_{Pa}}$ μA	$\Delta \bar{I_{Pa}}$ μA	$\mathrm{SD}\;\Delta \bar{I_{Pa}}$ μA	% SS	$\bar{I_{Pa}}$ μA	$\Delta \bar{I_{Pa}}$ μA	$\mathrm{SD}\;\Delta \bar{I_{Pa}}$	% SS
Blank		184.210				418.907			
CCP3 1	0	180.807	3.403	0.079	1.847	413.743	5.163	0.211	1.232
CCP3 10	1	179.047	5.163	0.081	2.803	397.100	21.807	0.237	5.205
CCP3 100	2	177.753	6.457	0.028	3.505	389.213	29.693	0.768	7.088
CCP3 1000	3	176.637	7.573	0.012	4.111	373.665	45.242	2.214	10.799
Blank		189.403				453.687			
C. *jejuni* 1	0	185.667	+3.737	0.168	1.979	451.050	2.637	0.228	0.581
C. *jejuni* 10	1	182.566	6.837	0.186	3.609	411.763	41.923	0.799	9.240
C. *jejuni* 100	2	175.173	14.230	0.579	7.513	388.050	65.637	4.092	14.467
C. *jejuni* 1000	3	171.146	18.257	0.872	9.639	362.207	91.480	2.654	20.163
Blank		187.457				406.940			
*C. fetus* 1	0	186.583	0.873	0.013	0.465	407.390	−0.450	0.012	−0.110
*C. fetus* 10	1	188.437	−0.980	0.022	−0.522	409.530	−2.590	0.066	−0.636
*C. fetus* 100	2	186.077	1.380	0.019	0.736	406.947	−0.007	0.001	−0.001
*C. fetus* 1000	3	189.273	−1.817	0.007	−0.969	409.787	−2.847	0.088	−0.699
Blank		188.063				456.460			
*E. coli.* 1	0	192.170	−4.107	0.057	−2.183	45.897	−3.437	0.061	−0.752
*E. coli* 10	1	193.313	−5.250	0.075	−2.791	461.310	−4.850	0.084	−1.062
*E. coli* 100	2	197.730	−9.667	0.548	−5.140	461.800	−5.340	0.089	−1.169
*E. coli* 1000	3	191.530	−3.467	0.054	−1.843	450.140	6.320	0.123	1.384
Blank		175.850				354.990			
*L. innocua* 1	0	173.733	2.117	0.084	1.203	354.100	0.890	0.063	0.250
*L. innocua* 10	1	174.783	1.067	0.053	0.606	350.480	4.510	0.359	1.270
*L. innocua* 100	2	174.513	1.337	0.071	0.760	355.717	−0.727	0.077	−0.205
*L. innocua* 1000	3	175.890	−0.040	0.002	−0.023	348.587	6.403	0.641	1.804
